# Computer-aided discovery of biological activity spectra for anti-aging and anti-cancer olive oil oleuropeins

**DOI:** 10.18632/aging.100691

**Published:** 2014-09-28

**Authors:** Bruna Corominas-Faja, Elvira Santangelo, Elisabet Cuyàs, Vicente Micol, Jorge Joven, Xavier Ariza, Antonio Segura-Carretero, Jordi García, Javier A. Menendez

**Affiliations:** ^1^ Metabolism & Cancer Group, Translational Research Laboratory, Catalan Institute of Oncology (ICO), Girona, Spain; ^2^ Girona Biomedical Research Institute (IDIBGI), Girona, Spain; ^3^ Departament de Química Orgànica, Fac. de Química, Institut de Biomedicina de la UB (IBUB), Universitat de Barcelona, Barcelona, Spain; ^4^ Instituto de Biología Molecular y Celular (IBMC), Universidad Miguel Hernández, Elche, Alicante, Spain; ^5^ Campus of International Excellence Southern Catalonia, Unitat de Recerca Biomèdica, Hospital Universitari de Sant Joan, Institut d'Investigació Sanitària Pere Virgili, Universitat Rovira i Virgili, Reus, Spain; ^6^ Department of Analytical Chemistry, Faculty of Sciences, University of Granada, Granada, Spain; Functional Food Research and Development Centre (CIDAF), PTS Granada, Granada, Spain

**Keywords:** PASS, Biological activity spectra, oleuropein aglycone, decarboxymethyl oleuropein aglycone, cancer, aging

## Abstract

Aging is associated with common conditions, including cancer, diabetes, cardiovascular disease, and Alzheimer's disease. The type of multi-targeted pharmacological approach necessary to address a complex multifaceteddisease such as aging might take advantage of pleiotropic natural polyphenols affecting a wide variety of biological processes. We have recently postulated that the secoiridoids oleuropein aglycone (OA) and decarboxymethyl oleuropein aglycone (DOA), two complex polyphenols present in health-promoting extra virgin olive oil (EVOO), might constitute anew family of plant-produced gerosuppressant agents. This paper describes an analysis of the biological activity spectra (BAS) of OA and DOA using PASS (Prediction of Activity Spectra for Substances) software. PASS can predict thousands of biological activities, as the BAS of a compound is an intrinsic property that is largely dependent on the compound's structure and reflects pharmacological effects, physiological and biochemical mechanisms of action, and specific toxicities. Using Pharmaexpert, a tool that analyzes the PASS-predicted BAS of substances based on thousands of “mechanism-effect” and “effect-mechanism” relationships, we illuminate hypothesis-generating pharmacological effects, mechanisms of action, and targets that might underlie the anti-aging/anti-cancer activities of the gerosuppressant EVOO oleuropeins.

## INTRODUCTION

The inter-species hormesis, or xenohormesis, hypothesis originally proposed by Dr. David A. Sinclair [1, 2] states that stress-induced synthesis of plant polyphenols and many other phytochemicals provides an environmental chemical signature that upregulates stress resistance pathways in plant consumers, including humans [1-5]. The existence of xenohormesis might explain how chemical compounds produced by plants and other autotrophs to defend against adverse environmental conditions can generate beneficial effects in the heterotrophs (animals and fungi) that consume them. Thus, we can take advantage of the healthy benefits that are chemically encrypted within plant-derived biocompounds. The natural polyphenolic compound resveratrol (3,5,4′-trihydroxystilbene) has emerged as an archetypal xenohormetic mediator of longevity that clearly delays or attenuates many age-related chronic diseases in animal models [6-12].

Dr. Mikhail V. Blagosklonny has recently proposed that “*hormesis does not make sense except in the light of TOR-driven aging*” [6]. In this scenario, aging-related disease (*e.g.,* atherosclerosis, diabetes, cancer, and other diseases) can be understood as the product of synergistic interactions between our evolutionary path to sedentarism, which increases a number of gero-promoting factors (*e.g.,* nutrients, growth factors, cytokines, insulin) that upregulate key gerogenes (*e.g.,* the nutrient-sensing mammalian target of rapamycin [mTOR]), and the “defective design” of central energy metabolism sensors that function either as metabolic gerogenes (*e.g.,* mTOR) or metabolic gerosuppressors (*e.g.,* AMP-activated protein kinase [AMPK], which antagonizes the gerogenic activity of mTOR) [13-29]. This “defective design” refers to (a) the ability of metabolic gerogenes to continue, in an aimless but harmful manner, a developmental program that was beneficial early in life but was not *switched off* upon its completion and therefore drives aging; and (b) the weakness of the metabolic gerosuppressors that antagonize the metabolo-gerogenic pathway. In this metabolic framework for aging-related diseases, upregulation of metabolic gerogenes limits lifespan by accelerating age-related diseases, whereas the responsiveness of metabolo-gerosuppressor signaling should decline with aging because robust and continuous activation of metabolic suppressors in response to metabolic stresses results in accelerated aging [30, 31]. In other words, the ability of metabolic gerogenes to drive aging can be triggered or accelerated by the loss of responsiveness of critical metabolic gerosuppressors to their appropriate activation.

If the upregulation of metabolic gerogenes limits lifespan by accelerating the progression of age-related diseases, such as atherosclerosis or cancer, the behavioral and/or pharmacological suppression of metabolic gerogene-driven aging (*e.g., via* non-permanent activation of metabolic gerosuppressors) should increase healthy lifespan. We have recently explored, for the first time, the putative AMPK/mTOR-related xenohormetic nature of complex polyphenols that are naturally present in extra virgin olive oil (EVOO), a pivotal component of the Mediterranean-style diet that has been repeatedly associated with a reduction in age-related morbidity and a longer life expectancy [29]. Using a crude EVOO phenolic extract that is highly enriched in the secoiridoids oleuropein aglycone (OA) and decarboxymethyl oleuropein aglycone (DOA), we have shown that EVOO oleuropeins, which provide an effective defense against the attack of plants by herbivores and pathogens, are *bona fide* xenohormetins that are able to activate the gerosuppressor AMPK and trigger numerous resveratrol-like anti-aging transcriptomic signatures in biologically aggressive cancer cells. As such, we postulated that EVOO secoiridoids constitute a new family of plant-produced gerosuppressant agents that molecularly “repair” the aimless (and harmful) AMPK/mTOR-driven quasi-program that leads to aging and aging-related diseases, including cancer [29, 32].

As a compound's biological activity spectrum (BAS) is an intrinsic property that is representative of different pharmacological effects, physiological and biochemical mechanisms of action, and specific toxicities, and the BAS is largely dependent on the structural nature of a compound [33-43], we recently hypothesized that computerized prediction of BAS might help us to elucidate hypothesis-generating pharmacological effects, mechanisms of action, and targets underlying the anti-aging/anti-cancer activity of gerosuppressant oleuropeins present in EVOO. Here, we employed Prediction of Activity Spectra for Substances (PASS) software [33-43] (www.pharmaexpert.ru/passonline/index.php), which is hosted by the V. N. Orechovich Institute of Biomedical Chemistry (www.ibmc.msk.ru/en) under the aegis of the Russian Foundation of Basic Research. Using Pharmaexpert (www.genexplain.com/pharmaexpert), a tool developed to analyze the BAS of substances predicted by PASS, we characterized EVOO oleuropeins with different types of biological activities based on multiple mechanisms of action, analyses of activity-activity relationships, and drug-drug interactions. We now report on how a multi-targeted pharmacological approach needed for a complex multifaceted disease, such as aging, might take advantage of EVOO-derived polyphenols, pleiotropically impacting a wide variety of biological processes.

## RESULTS

### Description of the chemical structures of EVOO oleuropeins

**Figure 1:**
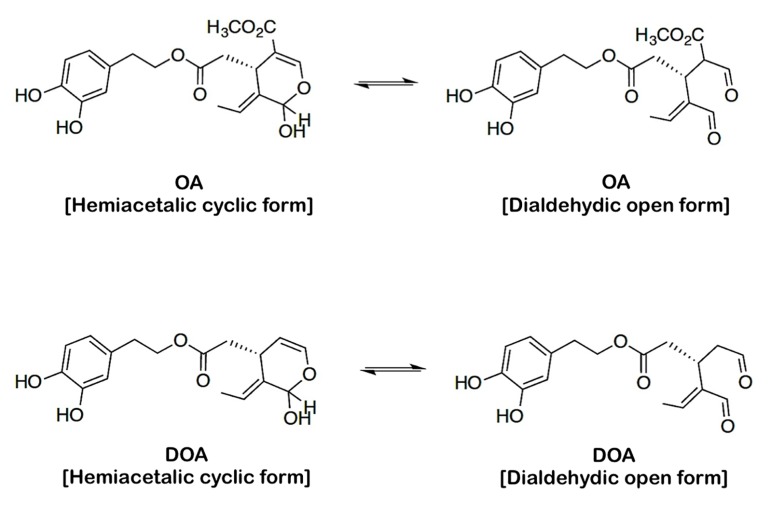
Structures of oleuropein aglycone (OA) and decarboxymethyl oleuropein aglycone (DOA)

OA and DOA are structurally related secoiridoids with identical dihydroxylated aromatic moieties that differ only due to the presence of a methoxycarbonyl group on C-5 of the dihydropyrane ring of OA (Fig. 1). As the PASS tool interprets the BAS based on the 2D structure of molecules, the hemiacetalic (“cyclic”) and dialdehidic (“open”) structures of OA and DOA were drawn using ACD/ChemSketch version 12, then saved as MDL Molfiles (*.mol) and directly uploaded into the PASS prediction program to predict the biologically active spectra of the molecules.

### PASS estimation of the BAS of EVOO oleuropeins

Predictions were made using the PASS12 refined version of the program (www.genexplain.com/pass), which predicts 1,105 types of biological activities with a mean prediction accuracy in leave-one-out cross-validation (LOOCV) of 96%. PASS prediction tools are constructed using principal compounds from the MDDR database (produced by Accelrys and Prous Science), which is continuously updated with biologically relevant compounds. The PASS training set consisted of 287,633 known biologically active substances (*e.g.,* drugs, drug-candidates, lead compounds, toxic compounds) compiled from various sources, including publications, patents, chemical databases, and “gray” literature [33-43].

**Figure 2:**
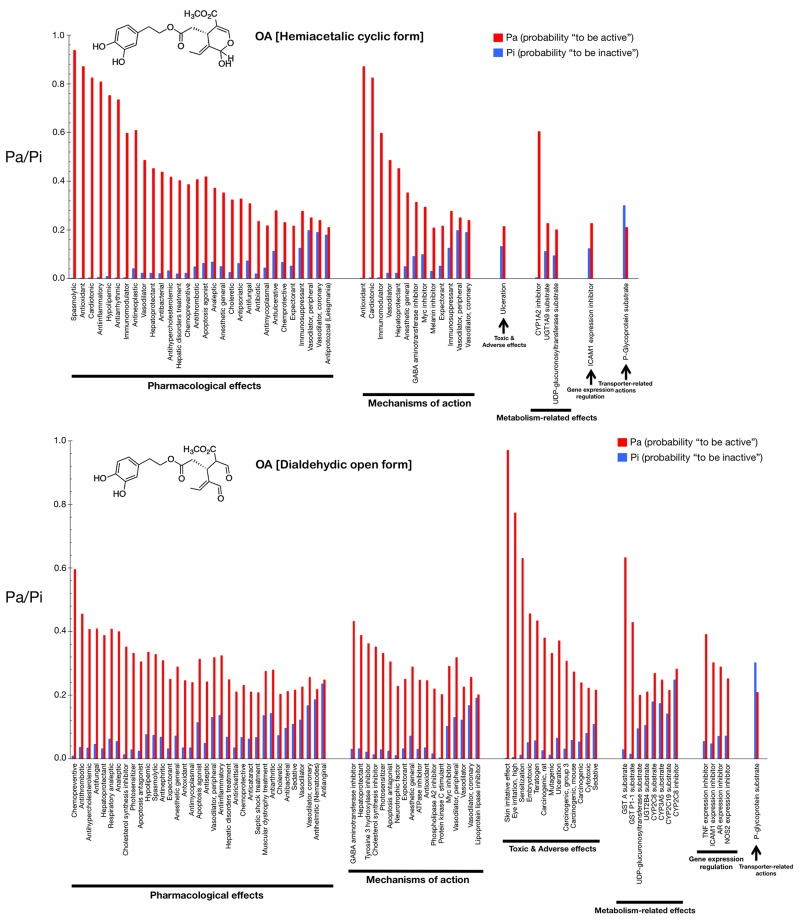
Biological activity spectra of the gerosuppressant olive oil oleuropein OA The results of predicted activity spectra generated by PASS are presented as a bar graph of biological activities with the probabilities “to be active” (Pa) and “to be inactive” (Pi) calculated for each activity. The values vary from 0.000 to 1.000; the higher a Pa value is the lower is the predicted probability of obtaining false positives in biological testing. The lists are arranged in descending order of Pa-Pi; therefore, more probable biological activities are at the top of the list. The list can be shortened at any desirable cutoff value, but PASS uses the criteria Pa=Pi as the as the default threshold, *i.e.,* only biological activities with Pa > Pi are considered as probable for a particular compound. If we choose to use rather high value of Pa as cutoff for selection of probable activities, the chance to confirm the predicted activities is high too, but many existing activities will be lost. For instance, if one selects for consideration particular biological activities predicted with Pa > 0.9, then about 90% of actual activities will be lost (*i.e.,* the expected probability to find inactive compounds in the selected set is very low but about 90% of active compounds will be missed). If one lowers the Pa threshold to 0.8, the probability to find inactive compounds is still low, but about 80% of active compounds will be missed, etc. Another important aspect of PASS predictions is the compounds' novelty. If one limits to high Pa values, one may find close analogues of known biologically active substances among the tested compounds. For instance, for Pa > 0.7, the chance to experimentally find the biological activity is high, but some of the activities may be close analogue of known pharmaceutical agents. If one chooses 0.5<Pa<0.7 values, the chances of obtaining activity in the experiment are lower, but the compound may be less similar to known pharmaceutical agents. For Pa < 0.5, the chances of obtaining activity in the experiment are even lower, but if activity is found, the compound might happen to be a new chemical entity. Nevertheless, it is important to keep in mind that the probability Pa reflects the similarity of a molecule under prediction with the structures of molecules, which are the most typical in a sub-set of “actives” in the training set. Therefore, there is no usually direct correlation between the Pa vales and quantitative characteristics of biological activities.

**Figure 3:**
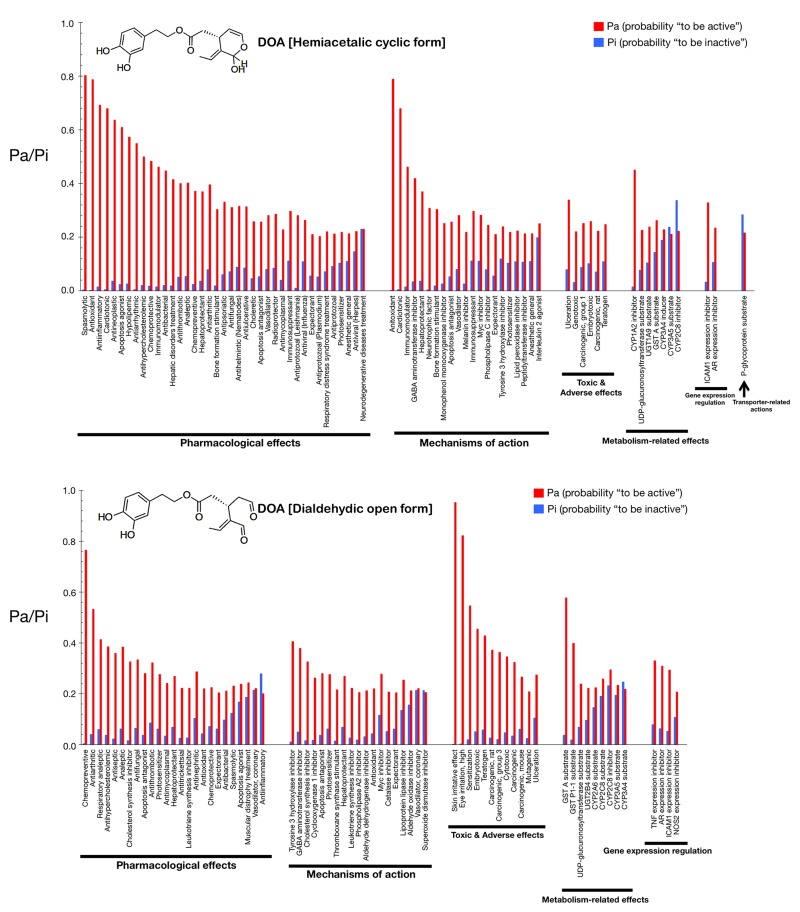
Biological activity spectra of the gerosuppressant olive oil oleuropein DOA (see Fig. 2 text for details).

Figs. 2 and 3 show only the activities predicted at a Pa (probability “to be active”) > 0.200 for the hemiacetalic and dialdehidic forms of OA (Fig. 2) and DOA (Fig. 3), grouped using PharmaExpert (www.genexplain.com/pharmaexpert), a tool that was developed to analyze the biological activity spectra of substances predicted by PASS. PharmaExpert software analyzes the relationships between biological activities (“mechanism-effect(s)” and “effect-mechanism(s)”), identifies probable drug-drug interactions, and searches for compounds acting on multiple targets [33-43].

Different types of biological activities are divided into six classes: *mechanisms of action*, *pharmacological effects*, *metabolism-related actions*, *transporter terms*, *gene expression terms*, and *toxic/adverse effects*.

The *mechanisms of action* reflect the interactions of biologically active compounds with biological entities at the macromolecular level. In addition to the expected antioxidant mechanism of action of OA (Pa = 0.872), PASS predicts some new mechanisms for the cyclic hemiacetalic form of OA, including cardiotonic (Pa = 0.826), immunomodulator (Pa = 0.598), vasodilator (Pa = 0.487), and hepatoprotectant (Pa = 0.453) activities. The cyclic hemiacetalic form of DOA is similarly predicted to exhibit an expected antioxidant mechanism of action (Pa = 0.790) while lacking all of the cardiotonic, immunomodulator, and hepatoprotectant mechanisms predicted for the cyclic hemiacetalic form of OA.

The open dialdehydic form of OA is predicted to lack all of the cardiotonic, immunomodulator, and hepatoprotectant mechanisms of action suggested for the cyclic hemiacetalic form of OA. Moreover, the expected antioxidant mechanism of OA is notably reduced when PASS predictions are used to estimate the probable mechanisms of action of the open dialdehydic form of OA (Pa = 0.247). The top predicted mechanism of action for the open dialdehydic form of OA is GABA aminotransferase inhibitor activity (Pa = 0.433). The open dialdehydic form of DOA is predicted to lack (Pa = 0.221) the highly predicted antioxidant mechanism of action of the cyclic hemiacetalic form of DOA, while gaining new mechanisms such as acting as a tyrosine 3 hydroxylase inhibitor (Pa = 0.407) and cholesterol synthesis inhibitor (Pa = 0.380).

The *pharmacological effects* reflect the pharmacological action or pharmacotherapeutic application of the compound. Some of the most likely anti-aging/anti-cancer pharmacological effects, showing higher Pa values in the predictions for the cyclic hemiacetalic form of OA, include anti-antioxidant (Pa = 0.872), anti-inflammatory (Pa = 0.809), anti-neoplastic (Pa = 0.610), apoptosis agonist (Pa = 0.419), and chemopreventive (Pa = 0.387) effects. The cyclic hemiacetalic form of DOA conserves all the anti-aging/anti-cancer pharmacological effects observed in the prediction for the cyclic hemiacetalic form OA, but displaying somewhat lower Pa values (*i.e.,* anti-oxidant [Pa = 0.790], anti-inflammatory [Pa = 0.693], and anti-neoplastic [Pa = 0.637] effects). The sole exception is the apoptosis agonist effect, which is predicted to occur with a higher probability (Pa = 0.610) for the cyclic hemiacetalic form of DOA.

Remarkably, the open dialdehydic form of OA exclusively conserves the chemopreventive effect of the cyclic hemiacetalic form of OA, and with an even higher Pa value (0.596). Similarly, the open dialdehydic form of DOA notably gains a strong anti-aging/anti-cancer-related chemopreventive effect (Pa = 0.766) compared with the weaker effect observed in the activity prediction for the cyclic hemiacetalic form of DOA (Pa = 0.372).

The *metabolism-related actions* reflect interactions of chemical compounds with metabolic enzymes. The cyclic hemiacetalic forms of OA and DOA are predicted to function as *CYP1A2* inhibitors (Pa = 0.605 and Pa = 0.451, respectively).

The open dialdehydic form of OA is predicted to function as a substrate of GST A and GST P1-1 (Pa = 0.633 and 0.430, respectively). Similarly, the open dialdehydic form of DOA is predicted to function as a GST A and GST P1-1 substrate (Pa = 0.579 and 0.400, respectively).

The *transporter terms* reflect the interaction of chemical compounds with transporters (*e.g.,* P-glycoprotein substrate, P-glycoprotein inhibitor, P-glycoprotein inducer). The cyclic and open forms of OA as well as the cyclic form of DOA are all predicted to function as P-glycoprotein substrates, but with a low Pa (~0.220); this activity is not predicted for the open dialdehydic form of DOA.

The *gene expression terms* reflect the influence of chemical compounds on the expression of certain genes. The cyclic hemiacetalic form of OA is predicted to negatively regulate *ICAM1* gene expression with a low Pa (0.228). The cyclic hemiacetalic form of DOA is similarly predicted to inhibit the expression of *ICAM1* (Pa = 0.329) and *AR* (Pa = 0.235) genes.

In addition to inhibiting the expression of the *ICAM1* gene (Pa = 0.303) and *AR* (Pa = 0.290), as indicated for the cyclic hemiacetalic form of OA, the open dialdehydic form is predicted to inhibit the expression of the *TNF* (Pa = 0.392) and *NOS2* (Pa = 0.253) genes. The open dialdehydic form of DOA, in addition to inhibiting the *ICAM1* (Pa = 0.295) and *AR* (Pa = 0.310) genes, as indicated for its cyclic hemiacetalic form, is also predicted to inhibit the expression of the *TNF* (Pa = 0.331) and *NOS2* (Pa = 0.208) genes.

The *toxic/adverse effects* reflect the specific toxicities or adverse reactions of the chemical compounds. No highly predicted toxic/adverse effects are indicated for the cyclic hemiacetalic form of OA beyond ulceration (Pa = 0.215). Interestingly, other indirectly related anti-aging/anti-cancer pharmacological effects with low Pa values are predicted for the cyclic hemiacetalic form of DOA, specifically embryotoxic (Pa = 0.260) and teratogen (Pa = 0.248) effects.

The open dialdehydic form of OA, in addition to showing skin irritation (Pa = 0.971) and eye irritation (Pa = 0.774) effects, is predicted to exhibit embryotoxic (Pa = 0.457) and teratogenic (Pa = 0.435) toxicities with Pa values that are somewhat higher than those predicted for its cyclic hemiacetalic form. A very similar pattern of toxic and adverse effects is predicted for the open dialdehydic form of DOA, including skin irritation (Pa = 0.954) and eye irritation (Pa = 0.823) effects as well as embryotoxic (Pa = 0.456), and teratogenic (Pa = 0.429) activities.

### Contributions of particular atoms to the anti-aging/anti-cancer activities of EVOO oleuropeins

**Figure 4:**
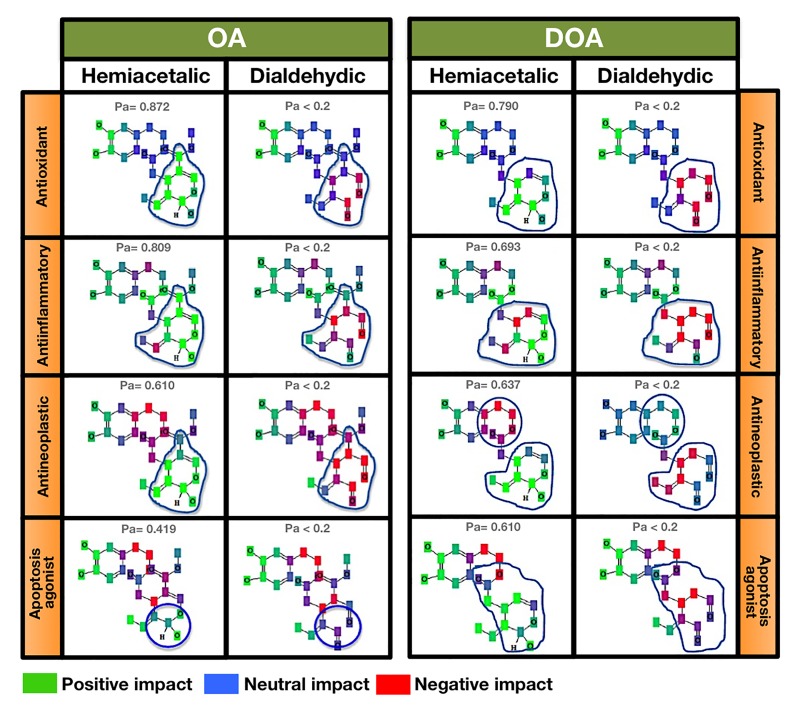
Contributions of particular atoms to the gerosuppressant activities of OA and DOA Figures provides a detailed comparison of the atomic groups that are likely to be responsible for the differences (blue circles) in the predictions of antioxidant, anti-inflammatory, antineoplastic, and apoptosis agonist activities with a Pa > 0.4 obtained for the hemiacetalic closed forms of OA and DOA that are not predicted (Pa < 0.2) for the dialdehydic open forms of OA and DOA.

To preliminarily elucidate hypothesis-generating structural requirements of gerosuppressant oleuropeins, we assessed how the naturally occurring chemical structures of OA and DOA (*i.e.,* the hemiacetalic closed forms versus dialdehydic open forms) impact some anti-aging/anti-cancer-related effects predicted with probabilities of Pa > 0.500 by PASS (*i.e.,* among the top 10 pharmacological effects predicted for each polyphenol isomeric form). Some of the pharma-cological effects assumed to be closely related to the previously reported anti-aging and anti-cancer effects of OA and DOA (*i.e.,* anti-oxidant, anti-inflammatory, and anti-neoplastic effects) are exclusively restricted to the hemiacetalic cyclic forms of OA and DOA (Fig. 4). The apoptosis agonist effect is predicted to occur with a notably higher probability in the cyclic hemiacetalic form of DOA (Pa = 0.610) than in the cyclic hemiacetalic form of OA (Pa = 0.419). Fig. 4 allows assessment of the impact of particular atoms on particular activities of the oleuropein isomers (**green** indicates a “positive impact”, **blue** a “neutral impact”, and **red** a “negative impact”). As green-colored atoms reflect a positive impact on a given activity while the red-colored atoms reflect a negative impact on the same activity, it should be noted that the chemopreventive mechanisms of action that are significantly predicted for the open dialdehydic forms of OA and DOA, but not for their cyclic hemiacetalic isomers mostly involve all of the atoms involved in the polyphenolic structure of DOA. Remarkably, as observed for many drugs with potential antineoplastic and chemopreventive properties, including curcumin and resveratrol [44-50], the open dialdehydic forms of OA and DOA are predicted to exhibit teratogenic and embryotoxic properties.

## DISCUSSION

Aging is associated with common conditions, including cancer, diabetes, cardiovascular disease, and Alzheimer's disease. A multi-targeted pharmacological approach necessary for addressing a complex multifaceted disease such as aging might take advantage of pleiotropic natural polyphenols affecting a wide range of biological processes. We recently postulated that the secoiridoids OA and DOA, two complex polyphenols present in health-promoting EVOO, might constitute a new family of plant-produced gerosuppressant agents [29, 32]. This paper describes, for the first time, the analysis of BAS for OA and DOA using PASS software.

The PASS algorithm is based on the concept of the BAS, which should be viewed as an intrinsic property of a compound. The BAS reflects all of the various biological activities that arise from the interactions of a compound with biological entities. Because in PASS, the biological activity spectrum represents a theoretical estimate for the general biological potential of the compound under study, this definition differs significantly from some other definitions of a “biological activity profile” or “biological activity spectrum” that are commonly published in the literature. PASS can then simultaneously predict pharmacological effects, mechanisms of action, mutagenicities, carcinogenicities, teratogenicities, and embryotoxicities based exclusively on the structural formula of a substance [33-43]. Using Pharmaexpert, a tool that analyzes the BAS of substances predicted by PASS based on a knowledgebase including thousands of “mechanism-effect(s)” and “effect-mechanism(s)” relationships, we have elucidated hypothesis-generating pharmacological effects, mechanisms of action, and targets that might underlie the anti-aging/anti-cancer activities of EVOO oleuropeins for the first time.

The results of the application of PASS to EVOO oleuropeins strongly support the notion that plant-derived dietary polyphenols may improve some disease states and promote health by operating as pleiotropic molecules that are capable of interacting with multiple molecular targets involved in aging and cancer-related processes, such as oxidation, inflammation, cholesterol and lipid synthesis, and immunosuppression. Although forthcoming studies should mechanistically confirm the predicted ability of the EVOO oleuropein DOA to exert anti-inflammatory, antihypercholesterolemic, and/or hepatoprotectant effects as part of the anti-aging and/or anti-cancer responses of DOA in *in vivo* models, we note that several studies have confirmed preventive effects of OA in experimental models of inflammation, inflammatory angiogenesis, and arthritis, in addition to hepatoprotective effects of OA in mice, chemopreventive effects in endothelial dysfunction-associated vascular diseases, and cardioprotective and neuroprotective effects [51-66].

Importantly, the results of our application of PASS to the EVOO oleuropeins OA and DOA, which exhibit two naturally occurring isomeric structures, strongly suggest that a different biological activity spectrum can be expected from hemiacetalic *versus* dialdehydic forms. In aqueous or physiological media, it should be assumed that both types of compounds exist in an equilibrium mixture consisting of a hemiacetal (*i.e.,* the cyclic form) and a dialdehyde isomer (*i.e.,* the open form). Although this equilibrium could be shifted in practice, for instance, by the preferential interaction of one of the isomers with a target protein, the dialdehyde form appears to be more conformationally free and adaptable to interact with a suitable target. It should be noted that the presence of the methoxycarbonyl constituent in OA clearly favors the cyclic hemiacetalic form due to the conjugation of the ester group with the internal double bond. In sharp contrast, a much greater ratio favoring the open dialdehydic form should be expected for DOA. Accordingly, the reported ^1^H-NMR of DOA shows that the dialdehyde form largely predominates [67-69]. In this regard, it is intriguing that application of PASS software to the isomeric forms of OA and DOA exclusively predicted (at least with high Pa values) chemopreventive activity for the open dialdehydic forms of OA, and more significantly, DOA. Chemopreventive agents derived from edible plants have been a part of the daily intake of many humans and animals since ancient times. There are multiple lines of compelling evidence from epidemiological, clinical, and laboratory studies that these dietary constituents are associated with a reduction of cancer risk [70-72]. It is reasonable to suggest that the gerosuppressant secoiridoids OA and DOA found in EVOO might be added to the list of plant-derived chemopreventive agents, not only based on their predicted antineoplastic effects, which have been unambiguously confirmed by our group in *in vitro* studies with cultured cancer cells, but also based on their predicted toxicities. Archetypal anti-aging/anti-cancer polyphenols, such as curcumin and resveratrol, have been shown to operate as “double-edged swords”, such that *in vitro* experiments reveal carcinogenic and pro-oxidant effects, in addition to anticancer and antioxidant effects. Accordingly, these polyphenols have been shown to exert embryotoxic and teratogenic effects using zebrafish embryos as disease models [44-50]. The PASS predictions obtained in the present study similarly predict carcinogenic, embryotoxic, and teratogenic effects for the dialdehydic forms of OA and DOA. Under the assumption that inhibition of developmentally regulated genes *in vitro* might also predict developmental toxicity under *in vivo* conditions [72], experiments are currently underway in our laboratory to examine the effect of the chemopreventive EVOO secoiridoid DOA on both the induction and differentiation of mouse induced pluripotent stem cells (iPSCs), as an *in vitro* model of embryotoxicity and the tumor-initiation properties of cancer stem cells.

We have recently postulated that the secoiridoids OA and DOA, two complex polyphenols present in health-promoting EVOO, might constitute a new family of plant-produced gerosuppressant agents [29, 32]. We demonstrated that the anticancer activity of EVOO phenolic extracts that were highly enriched in the secoiridoids OA and DOA was intriguingly related to the activation of anti-aging/cellular stress-like gene signatures (*e.g.,* associated with endoplasmic reticulum stress, the unfolded protein response, spermidine and polyamine metabolism, AMPK activation, suppression of crucial genes involved in the Warburg effect and the self-renewal capacity of “immortal” cancer stem cells), while the same OA- and DOA-enriched phenolic extracts significantly prevented age-related changes in cell size, morphological heterogeneity, and senescence-associated β-galactosidase staining of normal diploid human fibroblasts at the end of their proliferative lifespans [29]. Because the exploration of putative therapeutic mechanisms using conventional wet laboratory experiments to obtain a better understanding of the ultimate pharmacological behavior of these polyphenolic gerosuppressants was expected to be tedious, time consuming, and expensive, we were encouraged to develop *in silico* techniques. The results of the present study confirm that PASS software is extremely useful for the elucidation of unknown therapeutic mechanisms in plant-based drug discovery and, more importantly, reveals hypothesis-generating pharmacological effects, mechanisms of action, and targets that likely underlie the anti-aging/anti-cancer activities of complex polyphenols that are naturally present in EVOO.
